# Hepatitis B in Moroccan-Dutch: a quantitative study into determinants of screening participation

**DOI:** 10.1186/s12916-018-1034-6

**Published:** 2018-03-29

**Authors:** Nora Hamdiui, Mart L. Stein, Aura Timen, Danielle Timmermans, Albert Wong, Maria E. T. C. van den Muijsenbergh, Jim E. van Steenbergen

**Affiliations:** 1 National Coordination Centre for Communicable Disease Control, Centre for Infectious Disease Control, National Institute for Public Health and the Environment, Bilthoven, The Netherlands; 2 Department for Health Evidence, Radboud University Medical Center, Nijmegen, The Netherlands; 3 Department of Public and Occupational Health, Amsterdam Public Health research institute, VU University Medical Center, Amsterdam, The Netherlands; 4 National Institute for Public Health and the Environment, Bilthoven, The Netherlands; 5 Department of Statistics, Informatics and Mathematical Modelling, National Institute for Public Health and the Environment, Bilthoven, The Netherlands; 6 Pharos: Dutch Centre of Expertise on Health Disparities, Program Prevention and Care, Utrecht, The Netherlands; 7 Department of Primary and Community Care, Radboud University Medical Center, Nijmegen, The Netherlands; 8 Centre for Infectious Diseases, Leiden University Medical Centre, Leiden, The Netherlands

**Keywords:** Hepatitis B, Intention, Screening, Determinants, Moroccans, Netherlands, Random forest

## Abstract

**Background:**

In November 2016, the Dutch Health Council recommended hepatitis B (HBV) screening for first-generation immigrants from HBV endemic countries. However, these communities show relatively low attendance rates for screening programmes, and our knowledge on their participation behaviour is limited. We identified determinants associated with the intention to request an HBV screening test in first-generation Moroccan-Dutch immigrants. We also investigated the influence of non-refundable costs for HBV screening on their intention.

**Methods:**

Offline and online questionnaires were distributed among first- and second/third-generation Moroccan-Dutch immigrants using respondent-driven sampling. Random forest analyses were conducted to determine which determinants had the greatest impact on (1) the intention to request an HBV screening test on one’s own initiative, and (2) the intention to participate in non-refundable HBV screening at €70,-.

**Results:**

Of the 379 Moroccan-Dutch respondents, 49.3% intended to request a test on their own initiative, and 44.1% were willing to attend non-refundable screening for €70,-. Clarity regarding infection status, not having symptoms, fatalism, perceived self-efficacy, and perceived risk of having HBV were the strongest predictors to request a test. Shame and stigma, fatalism, perceived burden of screening participation, and social influence of Islamic religious leaders had the greatest predictive value for not intending to participate in screening at €70,- non-refundable costs. Perceived severity and possible health benefit were facilitators for this intention measure. These predictions were satisfyingly accurate, as the random forest method retrieved area under the curve scores of 0.72 for intention to request a test and 0.67 for intention to participate in screening at €70,- non-refundable costs.

**Conclusions:**

By the use of respondent-driven sampling, we succeeded in studying screening behaviour among a hard-to-reach minority population. Despite the limitations associated with correlated data and the sampling method, we recommend to (1) incorporate clarity regarding HBV status, (2) stress the risk of an asymptomatic infection, (3) emphasise mother-to-child transmission as the main transmission route, and (4) team up with Islamic religious leaders to help decrease elements of fatalism, shame, and stigma to enhance screening uptake of Moroccan immigrants in the Netherlands.

**Electronic supplementary material:**

The online version of this article (10.1186/s12916-018-1034-6) contains supplementary material, which is available to authorized users.

## Background

Hepatitis B (HBV) is one of the major infectious diseases in the world, which if chronic and untreated, has an increased risk for serious complications, such as liver cirrhosis and liver cancer [1]. In the Netherlands, 0.2% of the general population has a chronic HBV infection, and annually an estimated 200 individuals die from chronic sequelae [2].

Countries of African and Southeast Asian regions have the highest prevalence of chronic HBV [3]. Dutch population-based studies showed a significantly higher prevalence of chronic HBV infection in immigrants from all intermediate or high endemic countries [4, 5]. From these areas, the two largest immigrant groups in the Netherlands are Turks and Moroccans. In 2016, there were 397,471 Turkish-Dutch individuals and 385,761 Moroccan-Dutch individuals [6].

Two small studies showed hepatitis B surface antigen (HBsAg) prevalences for Moroccan-Dutch immigrants to be 0.4% (*n* = 281) and 0.0% (*n* = 50) [7, 8]. However, a systematic review and meta-analysis found similar chronic HBV prevalences for immigrants in Europe as those in their country of origin [9]. This would lead to an estimated HBsAg prevalence of 1.81% among Moroccan-Dutch immigrants [10], which is nine times higher compared to the HBV prevalence in the general Dutch population.

In November 2016, the Dutch Health Council recommended HBV screening for first-generation immigrants originating from intermediate or high HBV endemic countries with the aim of detecting chronically infected individuals for monitoring and immediate treatment if justified, hereby preventing further transmission. The predominant mode of transmission in the Moroccan population is mother to child at birth [1]. The Council proposed two screening strategies for first-generation immigrants: (1) individual case finding by general practitioners (GPs), and (2) local screening programmes in cities or regions with large numbers of first-generation immigrants originating from countries with intermediate or high endemicity [11]. Individual case finding suggests that not all patients visiting the GP will be consistently advised to test for HBV, but only high-risk individuals; the risk determination is based on — among other considerations — the country of origin. Therefore, it is important for the Moroccan-Dutch to know about this possibility to test and to enable them to request the test on their own initiative. Both screening strategies start with an HBV blood test, costing €35,- (2016). The Dutch health insurance is organised with a compulsory annual front-end deductible (“own risk”) of €385,- (2017). Therefore, the HBV blood test is not refundable for those for whom the threshold of €385,- has not yet been reached with other health care costs. The potential non-refundable costs of the test may inhibit the intention of Moroccan-Dutch individuals to test themselves for HBV.

Previous studies [12–15] have shown lower attendance rates among Moroccan-Dutch immigrants compared to indigenous populations for screening programmes involving breast and cervical cancer. In these studies, the most important determinants for non-participation were lack of awareness and knowledge, organisational issues, socio-cultural aspects (e.g. (health) illiteracy), perceived social norm, susceptibility, and benefits and barriers (e.g. fear of the test result). As it is unknown whether these determinants similarly influence participation in chronic HBV screening, we considered it essential to identify determinants of chronic HBV screening intention and to examine how screening can be promoted effectively in the Moroccan-Dutch community.

Therefore, our main objective was to identify determinants associated with the intention to actively request an HBV screening test (HBsAg blood testing) in first-generation Moroccan-Dutch immigrants. Since the potential costs of the screening test may discourage the Moroccan-Dutch to test themselves, we also investigated the intention of first-generation Moroccan-Dutch to participate in HBV screening for non-refundable costs of €70,-.

## Methods

### Study design

From November 2016 to February 2017, both offline-recruited and online-recruited respondents were enrolled in this study. Eligibility for participation was defined as (1) being aged 16 years or older, and (2) born in Morocco and having at least one parent born in Morocco (first-generation migrants, FGMs [16]) or born in the Netherlands and having at least one (grand)parent born in Morocco (second- or third-generation migrants, STGMs [16]), and (3) living in the Netherlands, and (4) not having participated in the study. The rationale for including STGMs was that they frequently act as brokers for their parents and grandparents in contact with the Dutch health care system. They usually also have a better command of the Dutch language and are more often found online [6]. Therefore, we asked STGMs and FGMs similar questions. STGMs were requested to answer the questions for their parents or grandparents as they thought suitable.

#### Respondent-driven sampling

We applied respondent-driven sampling (RDS) [17, 18], a variant of chain-referral sampling, to reach and distribute questionnaires among Moroccan immigrants in the Netherlands. RDS starts with a convenience sample of selected members of the target population. Respondents complete a questionnaire and are asked to invite “peers” from their social network to complete the same questionnaire as well. Using invitations containing unique codes, we registered who invited whom in order to follow the interactions within social networks for future analyses. We asked respondents to recruit three or more peers. We offered a gift coupon to both offline- and online-recruited respondents whenever someone successfully recruited three or more eligible persons of their network. The value of the gift coupon was gradually increased over time (in three steps: €5,-, €10,- and €25,-) to enhance peer recruitment. Online respondents could also see anonymous questionnaire results and their recruitment tree at the end of the questionnaire.

#### Offline recruitment

Offline-recruited respondents were asked to fill in a paper-based questionnaire, which was distributed in person or via paper mail. We invited respondents at community venues, such as community centres, day care centres, mosques, interest groups, and civil support foundations. Offline-recruited respondents could invite people both offline and online. Offline, respondents could choose between receiving paper questionnaires in person (if possible) or via paper mail. If online was preferred, respondents received a specified number of invitation messages containing a personal link via email or WhatsApp, which could be forwarded to others, enabling them to participate in the online questionnaire. Based on population numbers of 2004, first-generation Moroccan-Dutch immigrants mainly live in Amsterdam (21%), Rotterdam (12%), Utrecht (8%), and The Hague (8%) [19]. Some other (medium-sized) municipalities, including Gouda, Almere, Leiden, Haarlem, Eindhoven, and Tilburg, are also cities where relatively large numbers of Moroccans of the first generation live [19]. We therefore targeted these cities for the start of our offline recruitment.

#### Online recruitment

Online-recruited respondents were enrolled through advertisements on Moroccan-Dutch forums, Facebook, Instagram, the website of the Dutch National Institute for Public Health and the Environment (RIVM), and a Moroccan-Dutch website [20]. An online RDS questionnaire system, similar to the one developed by Bengtsson and colleagues [21], was used to apply online RDS. Recruiting peers online was enabled through indirect email (i.e. sending an email invitation to yourself, which could be forwarded to contacts), WhatsApp, Facebook, or by sharing a hyperlink.

### Study population

First-generation Moroccan-Dutch immigrants generally speak Berber and/or Arabic. As Berber languages and Arabic dialects are solely speaking languages, no written variant is available. Therefore, respondents were invited to complete a Dutch questionnaire. To reduce possible difficulties with reading Dutch, we used simple Dutch (B1 level). Whenever respondents wanted to invite someone who did not have a mastery of the Dutch language, they could provide this persons’ phone number in order for the researcher to contact this person to schedule a face-to-face or telephone interview in Berber. In the online questionnaire, we also provided audio recordings containing information about HBV, transmission, and testing in Dutch, Berber, and Arabic.

Respondents who reported to speak Berber were defined as Moroccan-Berber. A Moroccan-Arabic identity was identified whenever a respondent reported to speak Moroccan-Arabic and/or Modern Standard Arabic without the ability to speak Berber.

### Questionnaire

A questionnaire was developed and tested among both FGMs and STGMs (see Additional file 1). Items were based on formative qualitative research in which we discussed determinants originating from a compilation of the Health Belief Model (HBM), the Theory of Planned Behaviour (TPB), and Betancourt’s Model of Culture and Behaviour. This compilation was previously used in the Turkish-Dutch community [22] by van der Veen et al. This research group found it impossible to identify one model for potential determinants of HBV screening behaviour in this group. Therefore, we followed this approach. The HBM assumes that a subject is more likely to take a ‘health action’ whenever he perceives (1) the disease as serious, (2) himself susceptible to the disease, (3) benefits of the ‘health action’, (4) limited barriers to take the ‘health action’, (5) self-efficacy in relation to the ‘health action’, and (6) he receives a cue to take the ‘health action’ [23–25]. According to the TPB, intention reflects a person’s readiness to perform a certain health behaviour or action, explained by attitude, subjective norm, and perceived behavioural control [23, 26]. Betancourt’s Model of Culture and Behaviour is more specific, as it includes culture to explain its influence on health behaviours, either directly or through psychological processes [27].

Respondents without any knowledge or awareness of HBV were informed on the key characteristics of the virus, the disease, transmission, and testing, prior to completing the questionnaire. Detailed background information was made available in Dutch through our project website. The questionnaire included questions regarding socio-demographic factors (i.e. age, gender, country of birth, and educational level), relationship with the recruiter, social network size, knowledge about HBV, HBV vaccinating and testing history, stigma and shame regarding HBV, social influence, perceived susceptibility, self-efficacy, and severity of disease, intention to have an HBV blood test, and the perceived benefits and barriers of having this test. Additional file 1: Table S1 shows the set of outcome and predictor variables included in the questionnaire. In the Netherlands, individuals without other health care costs have to pay €35,- (in 2017) for laboratory tests used in screening. In the questionnaire, we defined the maximum non-refundable costs at €70,- to take a possible future cost increase into account.

### Statistical analysis

Descriptive analyses were conducted for the total group, for first-generation, and for second/third-generation Moroccan-Dutch immigrants. For our multivariate analyses, we used random forest (RF). RF is a machine learning method that uses a non-parametric algorithm to predict an outcome and to select important determinants. RF is appropriate here, as our questionnaire consisted of a large number of possible determinants relative to the number of respondents, which leads to a high risk of overfitting and false positives (in the context of identifying important variables). Previous studies have also shown a favourable performance of RF in comparison to other variable selection methods, including those that are related to the often-used logistic regression [28, 29]. The RF method yields a convenient ranking of variables in terms of how predictive they are in relation to the outcome (see Additional file 1), the so-called variable importance ranking. The predictability of variables is determined through the mean decrease in accuracy. The more the accuracy of the RF model decreases by excluding a single variable, the more important the variable. Therefore, variables with a large mean decrease in accuracy are deemed more relevant for classification of the data. The RF method can also be used (as is the case with most methods) to estimate so-called marginal probabilities for a given variable. We defined a marginal probability as the average model-based probability over all individuals, given that they assume a certain value for that variable whilst holding all other variables constant at their original values (as is observed in the sample).

First, RF analyses were done with ‘intention request’ as the dependent variable and all possible determinants as independent variables, as depicted in Additional file 1: Table S1. This intention measure represents the intention for requesting an HBV test on one’s own initiative. Second, to investigate the influence of having to pay for screening, we repeated the RF analyses using the outcome measure ‘intention to participate in HBV screening for non-refundable costs of €70,-’ as the dependent variable. We will further refer to this outcome measure as ‘intention 70’. All possible determinants (Additional file 1: Table S1) were again included as independent variables.

Initially, we built two RF models, one with ‘intention request’ as the dependent variable and another one with ‘intention 70’. These models were trained using a subset of the individuals who responded to *all* 33 variables. A ten times repeated tenfold cross-validation was performed to gauge the RF models’ performance [30]. Furthermore, a restricted forward feature selection [31] was used to determine how many variables are relevant for predicting the outcome [31]. The selection procedure involved adding variables one by one, each time checking the model’s performance. The number of relevant variables should correspond with the point at which a (strong) improvement in the model’s performance no longer can be seen. The order in which variables are added follows the aforementioned variable importance ranking, i.e. starting with the single most important variable and subsequently including less important variables one by one. Subsequently, we again built two RF models, each with its own dependent variable (‘intention request’ and ‘intention 70′) and the previously determined number of most important variables with their confusion matrices. The confusion matrix depicts the number of true positives (TPs), true negatives (TNs), false positives (FPs), and false negatives (FNs), classified using the training data. Model performance was gauged by checking the model’s classification accuracy (ACC), sensitivity (SENS), specificity (SPEC), and the area under the curve (AUC) (see Additional file 1). In Additional file 1, we also described the total RF model results for ‘intention request’ and the complete RF results for ‘intention 70’. Furthermore, we investigated the influence of missing values on our main results by including missing values as a separate category (to increase the amount of analysable data). We decided not to use imputation, since (1) it has not been studied well for RF and it has never been shown that it is better than defining missing values as a separate category, and (2) for RF only single imputations are involved, which we found highly undesirable considering that the uncertainty of the imputation is not taken into account. Statistical analyses were conducted using R version 3.2.0. To perform RF, the “randomForest” and “caret” packages were used.

## Results

### Sample characteristics

In total, we invited 350 Moroccan-Dutch immigrants, of which 143 (40.9%) were invited offline and 207 (59.1%) were invited online. Of those 350 invited individuals, 242 participated (response rate of 69.1%) in the study. These individuals recruited another 165 recruits, which resulted in 407 respondents (see Table 1). Respondents consisted of 193 (50.9%) first-generation Moroccan-Dutch immigrants (FGMs), 186 (49.1%) second- or third-generation Moroccan-Dutch immigrants (STGMs), 8 (2.0%) were born neither in the Netherlands nor in Morocco, and 20 (4.9%) had an unknown country of birth. The latter two groups of respondents were excluded, which led to a total sample of 379 Moroccan-Dutch respondents. Of these, 135 (35.6%) reported a higher educational level, 172 (45.4%) secondary school or vocational education, and 66 (17.4%) indicated no official education or primary school. Of the total sample, 79 (20.8%) self-reported to be already tested for HBV and 115 (30.3%) reported to be vaccinated against HBV.

**Table 1:** Demographics and testing characteristics of Moroccan-Dutch immigrants

Characteristic		First generation (*n* = 193, 50.9%)	Second or third generation (*n* = 186, 49.1%)	Total (*n* = 379)
Offline/online participation	Offline	110 (57.0)	46 (24.7)	156 (41.2)
	Online	83 (43.0)	140 (75.3)	223 (58.8)
	*Missing value*	0 (0)	0 (0)	0 (0)
Moroccan-Arabic or Berber identity	Arabic	70 (36.3)	81 (43.5)	151 (39.8)
	Berber	122 (63.2)	105 (56.5)	227 (59.9)
	*Missing value*	1 (0.5)	0 (0)	1 (0.3)
Gender	Male	64 (33.2)	59 (31.7)	123 (32.5)
	Female	129 (66.8)	127 (68.3)	256 (67.5)
	*Missing value*	0 (0)	0 (0)	0 (0)
Age group	16–25 years	2 (1.0)	84 (45.2)	86 (22.7)
	26–35 years	18 (9.3)	59 (31.7)	77 (20.3)
	36–45 years	58 (30.1)	36 (19.4)	94 (24.8)
	46–55 years	63 (32.6)	1 (0.5)	64 (16.9)
	56–65 years	30 (15.5)	1 (0.5)	31 (8.2)
	66 years and older	12 (6.2)	2 (1.1)	14 (3.7)
	*Missing value*	10 (5.2)	3 (1.6)	13 (3.4)
Educational level	No official education or primary school	62 (32.1)	4 (2.2)	66 (17.4)
	Secondary school	31 (16.1)	44 (23.7)	75 (19.8)
	Vocational education	44 (22.8)	53 (28.5)	97 (25.6)
	Higher education	51 (26.4)	84 (45.2)	135 (35.6)
	*Missing value*	5 (2.6)	1 (0.5)	6 (1.6)
Speaking Dutch (SR)	Yes	181 (93.8)	185 (99.5)	366 (96.6)
	No	11 (5.7)	1 (0.5)	12 (3.2)
	*Missing value*	1 (0.5)	0 (0)	1 (0.3)
Knowledge on HBV	No	79 (40.9)	73 (39.2)	152 (40.1)
	Limited	82 (42.5)	80 (43.0)	162 (42.7)
	Sufficient	32 (16.6)	33 (17.7)	65 (17.2)
	*Missing value*	0 (0)	0 (0)	0 (0)
HBV in family or friends	Yes	52 (26.9)	25 (13.4)	77 (20.3)
	No	119 (61.7)	135 (72.6)	254 (67.0)
	I do not know	22 (11.4)	26 (14.0)	48 (12.7)
	*Missing value*	0 (0)	0 (0)	0 (0)
Tested for HBV (SR)	Yes	43 (22.3)	36 (19.4)	79 (20.8)
	No	128 (66.3)	131 (70.4)	259 (68.3)
	I do not know	21 (10.9)	19 (10.2)	40 (10.6)
	*Missing value*	1 (0.5)	0 (0)	1 (0.3)
Vaccinated against HBV (SR)	Yes	55 (28.5)	60 (32.3)	115 (30.3)
	No	60 (31.1)	48 (25.8)	108 (28.5)
	I do not know	78 (40.4)	78 (41.9)	156 (41.2)
	*Missing value*	0 (0)	0 (0)	0 (0)
Intention request	Yes/probably yes	100 (51.8)	87 (46.8)	187 (49.3)
	No/probably not	83 (43.0)	85 (45.7)	168 (44.3)
	*Missing value*	10 (5.2)	14 (7.5)	24 (6.3)
Intention 70	Yes/probably yes	83 (43.0)	84 (45.2)	167 (44.1)
	No/probably not	91 (47.2)	85 (45.7)	176 (46.4)
	*Missing value*	19 (9.8)	17 (9.1)	36 (9.5)

Data are reported as number of respondents (%)

*SR* Self-reported

When excluding all missing values for RF analyses, 306 and 303 respondents were included in the model with ‘intention request’ and ‘intention 70’, respectively

Of the FGMs, 83 (43.0%) reported having a negative intention to request an HBV test on their own initiative (‘intention request’). Furthermore, 91 (47.2%) reported having a negative intention to participate in HBV screening for a maximum own contribution of €70,- (‘intention 70’). Of the STGMs, 85 (45.7%) and 85 (45.7%) reported having a negative ‘intention request’ and ‘intention 70’, respectively.

### Multivariate associations for ‘intention request’

**Fig. 1:**
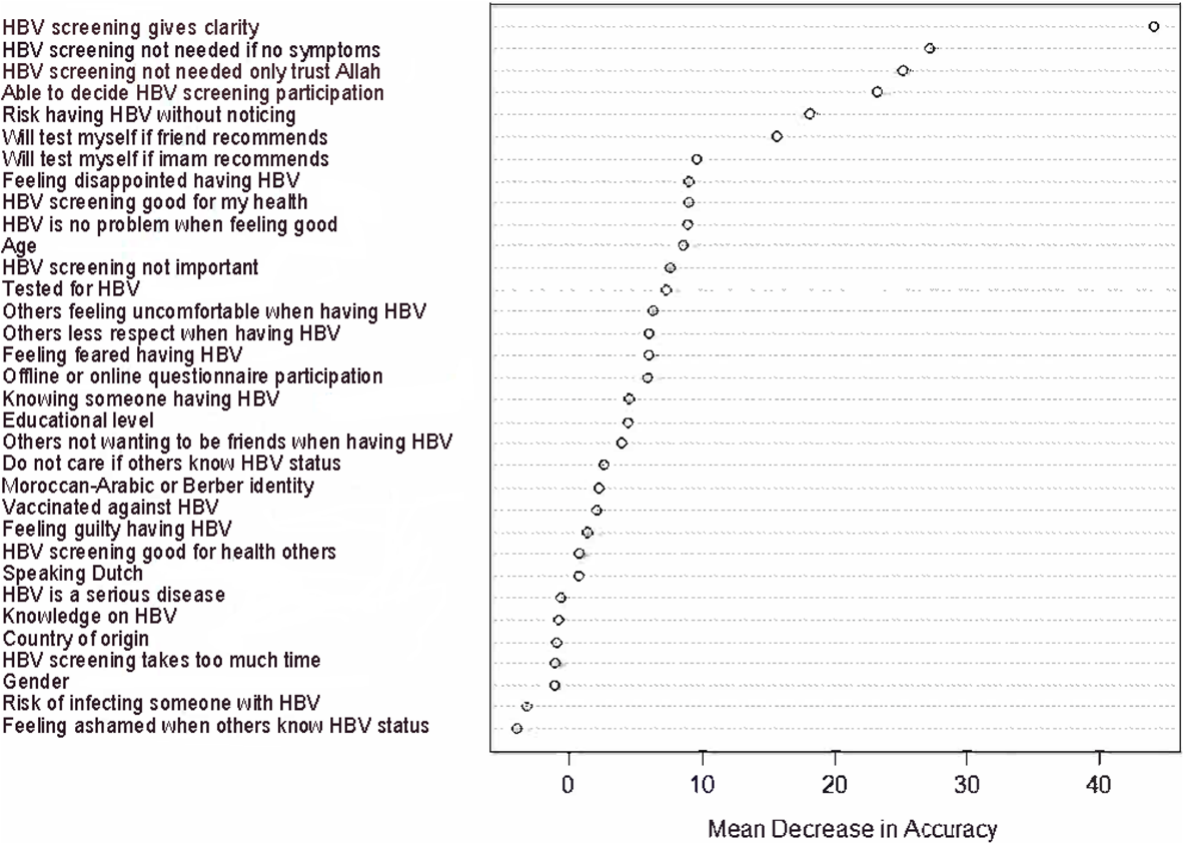
Variable importance analysis performed by RF for ‘intention request’ (*n* = 306). The set of 33 variables used for classification, ordered by their mean decrease in accuracy (importance) as estimated by RF

The RF model with ‘intention request’ and 33 predictor variables obtained an AUC of 0.681 (see Additional file 1: Table S2). Multivariate associations to determine variable importance for ‘intention request’ yielded five top predictors for requesting a test, which were ‘benefit clarity’, ‘barrier not having symptoms’, ‘barrier trusting Allah’, ‘self-efficacy’, and ‘risk without noticing’ (see Fig. 1).

**Fig. 2:**
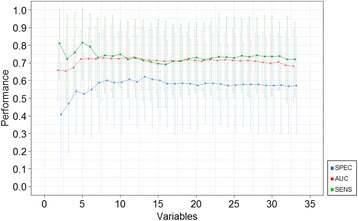
Result of restricted forward feature selection with RF model for ‘intention request’. This figure shows the AUC, SENS, and SPEC for ‘intention request’ starting with the most important variable and adding each variable one by one to the model, following the rank obtained through calculating the mean decrease in accuracy (displayed in Fig. 1)

The RF model for ‘intention request’ achieved the peak AUC value (0.722) after including the five most important variables (see Table 2 for the confusion matrix). Including more variables had a negligible effect (see Fig. 2).

**Table 2:** Performance of the RF model for ‘intention request’ with the top five variables

		Observed intention	
		Positive intention	Negative intention
Predicted intention by RF	Positive intention	132 (43.1%)	30 (9.8%)
	Negative intention	68 (22.2%)	76 (24.8%)

Data in this confusion matrix are presented as the numbers and percentages of observed and predicted respondents to have a positive or negative intention according to RF

Performance metrics: ACC 0.680 (standard deviation, SD 0.116); AUC 0.722 (SD 0.080); SENS 0.815 (SD 0.105), and SPEC 0.525 (SD 0.115)

Relative to each other, ‘benefit clarity’, ‘barrier not having symptoms’, ‘barrier trusting Allah’, perceived self-efficacy, and perceived risk showed distinctive estimated marginal probabilities for having a positive ‘intention request’ (see Table 3). For example, respondents who stated ‘participating in HBV screening will give me clarity’ (i.e. a decisive answer) had a marginal probability of 0.541 of requesting a test, whilst respondents who answered negative on this statement had a marginal probability of 0.327 of not requesting a test. This corresponded well with the marginal probability of having a positive intention for respondents who stated ‘I do not know’ for their perceived risk of having HBV without noticing (0.585). In this regard, respondents who did not know their risk seemed to desire clarity regarding their HBV status and indicated to be willing to request an HBV test.

**Table 3:** Marginal probabilities of the top five variables in relation to ‘intention request’

Variables	Content	Answering options	Marginal probability
Benefit clarity	Participating in HBV screening will give me clarity (i.e. a decisive answer)	Yes	0.541
		No	0.327
		I do not know	0.331
Barrier not having symptoms	Participating in HBV screening is not needed if I do not have symptoms or complaints	Yes	0.412
		No	0.555
		I do not know	0.498
Barrier trusting Allah	Participating in HBV screening is not needed as I only trust Allah	Yes	0.464
		No	0.547
		I do not know	0.512
Self-efficacy	I think I am able to decide whether or not to participate in HBV screening	Yes	0.538
		No	0.429
		I do not know	0.520
Risk without noticing	Perceived risk of having HBV without noticing	Low	0.475
		Quite low	0.549
		Average	0.568
		Quite high	0.563
		High	0.576
		I do not know	0.585

### Multivariate associations for ‘intention 70’

The total RF model with ‘intention 70’ retrieved an AUC of 0.638. The top five predictors of the willingness to attend non-refundable screening for €70,- were ‘shame others’, ‘barrier trusting Allah’, ‘barrier too much time’, ‘offline or online questionnaire participation’, and ‘stigma comfort’. However, Additional file 1: Figure S2 shows that the RF model for ‘intention 70’ was most predictive by including the ten most important variables. The final RF model with the ten most important variables yielded an AUC of 0.666. Additional file 1: Table S5 shows that respondents who stated ‘I would feel ashamed if I have HBV and others would know this’ had an estimated marginal probability of 0.420 of having a positive ‘intention 70’, whilst respondents who answered negative on this statement had a marginal probability of 0.509 of having a positive ‘intention 70’.

## Discussion

This is the first study, to our knowledge, that investigates hepatitis B screening behaviour among Moroccan-Dutch immigrants. We found that clarity regarding HBV status, not having symptoms or complaints, fatalism (i.e. an attitude emphasising the subjugation of all events to fate), high level of perceived self-efficacy, and perceived risk of having HBV were the strongest predictors to actively request an HBV test among the Moroccan-Dutch. This information is important for the development of future HBV screening promotion in the Moroccan-Dutch community.

In our study, shame and stigma regarding HBV, fatalism, perceived burden of participating in screening, perceived severity, social influence of the imam (i.e. Islamic religious leader), and the possible health benefit had the greatest predictive value for the intention to participate in screening for a maximum own contribution of €70,-. By using both offline and online RDS, we surveyed different individuals with different intentions to participate in screening. Offline participants (predominantly elderly) were more willing to test for HBV in comparison to those who participated online.

The large number of relevant predictors indicates a complex and diverse determination of the intention to participate in HBV screening in Moroccan-Dutch inhabitants. It was expected that knowledge of HBV would be one of the strongest predictors, as reported in previous studies on cancer screening programmes [12, 32–34]. However, our data did not support this finding. This might be explained by the low percentage of individuals having sufficient knowledge before starting the questionnaire, prohibiting analyses of associations of knowledge with intention. Moreover, before respondents were surveyed on their HBV perceptions, we eliminated knowledge as a discernible determinant, as we had to bring all respondents to the same minimal knowledge level to enable participation in the questionnaire. Furthermore, we have seen that 20.8% thought that they had already been tested for HBV and 30.3% reported to be vaccinated against HBV. We seriously question the truthfulness of these reported data, as most of the respondents had no or insufficient knowledge on HBV prior to our introduction, and without sufficient knowledge it is difficult to discern blood tests or vaccinations according to causative agents. All travellers to Morocco are advised to take protection against viral hepatitis A. This might easily have caused hepatitis recall difficulties. We repeated our analysis for a sample excluding respondents who reported to be already tested or vaccinated against HBV, and we found similar results for both intention measures. The most important variables were identical; less important ones differed slightly (data not shown).

### Comparison with other studies

To date, no similar research on hepatitis B screening intention has been conducted among Moroccans in the Netherlands, Morocco, or other countries. Therefore, we can only compare our study with studies on the intention of Moroccan-Dutch to participate in breast and cervical cancer screening [12, 32, 33, 35–37]. In contrast to our study, a narrative literature review indicated lack of knowledge about examination, fear or shame of (results of) examination, not having received or understood the (Dutch) invitation letter, and lack of satisfaction with the GP as inhibitors within the Turkish- and Moroccan-Dutch community [12]. As mentioned, we were unable to study knowledge as a determinant. De Nooijer et al. (2005) showed a higher participation rate in women born in Morocco after an invitation by the GP compared to an invitation by the Municipal Public Health Service (MPHS) [35]. We have not explored how participation rate is affected by the organisation responsible for the invitation, since the Dutch Health Council advised to organise individual case finding through GPs. In Denmark, perceived severity, perceived risk, and lack of emotional support were found to be associated with screening participation among migrant women, and these results are in accordance with our study [33, 36]. Similar determinants were found in Moroccan-Spaniards [34, 38]. A Moroccan study reported room for improvement when it comes to knowledge of breast cancer risk factors in female health care professionals in Morocco [32]. Furthermore, a pilot cervical cancer screening programme in Morocco acquired a low compliance rate of 6.0% in 2011–2013, which was explained by the lack of a mass communication and awareness campaign regarding the screening programme [37].

Compared to the Moroccan-Dutch, there is considerably more knowledge for the Turkish-Dutch population on determinants for participation in chronic hepatitis B screening. Despite several differences between Moroccan-Dutch and Turkish-Dutch immigrants, such as culture, Dutch language proficiency, and screening participation, we thought it is wise to compare these two groups because of their comparable migration status and religion. A study on the intention to participate in HBV screening in the Turkish-Dutch population identified perceived behaviour control and subjective norm of the TPB as the strongest predictors [22]. Perceived behaviour control was explained by shame and stigma regarding HBV and associating HBV screening with sexuality, and subjective norm was explained by family values. We also found shame and stigma regarding HBV and social influence of the imam as strong predictors for ‘intention 70’. However, this was not true for ‘intention request’, which can be explained by the fact that van der Veen et al. [22] only asked for the intention to participate in HBV screening and not the intention to request a test on one’s own initiative. We also have taken the influence of cost into account and included the GP as health care provider, in accordance with the Dutch Health Council’s advice, and not the MPHS as van der Veen et al. did.

### Strengths and limitations

For the first time, we can report on important determinants for intention to participate in HBV screening among the Moroccan-Dutch population. Second, offline, we targeted the four big cities (Amsterdam, Rotterdam, Utrecht, and The Hague) and some other (medium-sized) municipalities, such as Leiden and Tilburg, where large numbers of Moroccans of the first generation live, and for which the Dutch Health Council also proposed local HBV screening programmes. Third, by using RDS, we were able to reach 379 respondents in only 3 months, which is a high number of respondents considering the challenges that come with conducting studies among migrant populations, and was higher than the number of respondents included in the single other similar study among the Turkish-Dutch population [22]. Finally, we used a combined theoretical model to detect all potential predictors within the Moroccan-Dutch community.

However, a number of limitations should also be addressed. A larger percentage of respondents were female (67.5%) and reported a high(er) educational level (35.6%), compared to what was observed in the 2015 sample by Statistics Netherlands [39], which may have caused selection bias. This bias is likely to be mitigated by including education and gender as potential confounders in our models. Second, there was a moderate degree of model uncertainty, as evidenced by the large standard deviations of the ACC, AUC, SENS, and SPEC. Nevertheless, our models yielded AUC scores (0.722 and 0.666, respectively) that were still considerably higher than 0.5 (which corresponds with random guessing). Third, RDS leads to data that are correlated between respondents, whilst independence of data is one of the assumptions of RF. However, we are not aware of any machine learning approach that can deal with correlated observations, and we argue that the application of RF to such data can still yield some strong clues as to which factors are important determinants. Furthermore, RDS helped us to reach this so-called “hard-to-reach minority population” successfully, which would have been much more challenging through more traditional random sampling strategies. Fourth, missing data were not imputed, and this may have introduced bias. Investigating the influence of missing values on our main results by including missing values as a separate category yielded similar findings for ‘intention request’ (i.e. identical top predictors but slightly different other predictors) (data not shown). However, doing the same for ‘intention 70’ led to different results (see Additional file 1: Figure S3), as it resulted in only four of the ten identical top variables (‘shame others’, ‘barrier too much time’, ‘shame guilty’, and ‘social influence imam’). The model’s prediction accuracy and its standard deviation were not affected much by including missing values as a category. Finally, our study had several risk factors for respondents waving or refusing participation, such as language barriers and HBV-associated shame and stigma. To overcome these factors, we helped respondents to complete the questionnaire through a face-to-face or telephone interview (offline-recruited respondents) and audio recordings in Dutch, Berber, and Moroccan-Arabic (online-recruited respondents), and we focused the questionnaire on the predominance of mother-to-child HBV transmission.

### Implications and future research

In planning communication strategies targeting the Moroccan-Dutch for HBV screening, we recommend emphasising ‘getting clarity regarding HBV status by participating in screening’ in information leaflets and oral information aimed at Moroccan-Dutch immigrants. In an educational campaign aiming to increase the knowledge on HBV, it is also important to stress the risk of having chronic hepatitis B despite feeling healthy. The most important predictors for non-participation in ‘intention 70’ were shame and stigma regarding HBV. In the Netherlands, HBV is mainly transmitted sexually and is classified as a sexually transmitted disease [40]. Dutch preventive programmes focus on men having sex with men and people who inject drugs, which may indeed lead to feelings of shame and stigma, as 97% of Moroccan-Dutch immigrants are Muslim, prohibiting both practices [6]. In the Moroccan epidemiology of HBV, the perinatal transmission dominates, and practically all chronically infected Moroccan-Dutch acquired their infection at birth without any relation to homosexual activity or intravenous drug use. It is therefore essential to emphasise the predominant transmission route of mother to child in an educational campaign.

Fatalism was shown to be an important predictor for both ‘intention request’ and ‘intention 70’. Therefore, Islamic religious leaders should, in our opinion, inform Muslims in mosques that Islam also advocates health-promoting activities and recommends those who are ill or are at risk of getting ill to strive to do anything to recover or prevent disease. These leaders would not only communicate information, helping to decrease elements of fatalism, but would also help decrease elements of shame and stigma and so increase acceptance.

Finally, before developing and implementing HBsAg screening methods directed at Moroccan-Dutch immigrants, it would be wise to pilot these in combination with actual screening to quantify the actual risk of chronic hepatitis in this population.

## Conclusions

To enhance screening uptake of Moroccan-Dutch immigrants, promoting activities should (1) incorporate clarity regarding HBV status, (2) stress the risk of an asymptomatic infection, (3) emphasise mother-to-child transmission as the main transmission route, and (4) team up with Islamic religious leaders to help decrease elements of fatalism, shame, and stigma.

## Additional file

Additional file 1: Supplementary information: ‘Random forest’, ‘Interpreting confusion matrices’, and ‘Used questionnaire’. Supplementary tables and figures: **Table S1.** Overview of variables measured by the questionnaire. **Table S2.** Performance of the total RF model with ‘intention request’; confusion matrix. **Table S3.** Performance of the total RF model with ‘intention 70’; confusion matrix. **Figure S1.** Variable importance analysis performed by RF for ‘intention 70’ (*n* = 303). **Figure S2.** Result of restricted forward feature selection with RF for ‘intention 70’. **Table S4.** Performance of the RF model with the top 10 variables for ‘intention 70’; confusion matrix. **Table S5.** Marginal probabilities of the top 10 variables in relation to ‘intention 70’. **Figure S3.** Variable importance analysis performed by RF for ‘intention 70’, including missing values as category (*n* = 379). **Table S6.** Performance metrics of all RF models. (DOCX 686 kb)

## Abbreviations

ACC: Model’s classification accuracy
AUC: Area under the curve
FGM: First-generation migrant
GP: General practitioner
HBM: Health Belief Model
HBsAg: Hepatitis B surface antigen
HBV: Hepatitis B virus
MPHS: Municipal Public Health Service
RDS: Respondent-driven sampling
RF: Random forest
RIVM: Dutch National Institute for Public Health and the Environment
SENS: Sensitivity
SD: Standard deviation
SPEC: Specificity
STGM: Second- or third-generation migrant
TPB: Theory of Planned Behaviour
