# Supplementary material for: Hepatitis B in Moroccan-Dutch: a quantitative study into determinants of screening participation
Source: BMC Med. 2018 Mar 29;16:47. doi: 10.1186/s12916-018-1034-6 (PMC5877391; doi:10.1186/s12916-018-1034-6)
Supplement: Supplementary file 1 — Supplementary information: ‘Random forest’, ‘Interpreting confusion matrices’, and ‘Used questionnaire’. Supplementary tables and figures: Table S1. Overview of variables measured by the questionnaire. Table S2. Performance of the total RF model with ‘intention request’; confusion matrix. Table S3. Performance of the total RF model with ‘intention 70’; confusion matrix. Figure S1. Variable importance analysis performed by RF for ‘intention 70’ (n = 303). Figure S2. Result of restricted forward feature selection with RF for ‘intention 70’. Table S4. Performance of the RF model with the top 10 variables for ‘intention 70’; confusion matrix. Table S5. Marginal probabilities of the top 10 variables in relation to ‘intention 70’. Figure S3. Variable importance analysis performed by RF for ‘intention 70’, including missing values as category (n = 379). Table S6. Performance metrics of all RF models. (DOCX 686 kb) [file 12916_2018_1034_MOESM1_ESM.docx]

**Supplementary information**

**Random forest**

Random forest (RF) is a non-parametric method that uses a set of decision trees to make predictions in regression and classification problems, and to select important variables [1]. In this study, we used RF for selecting variables in a classification context. Each tree is grown by taking a bootstrap sample of the training data, selecting a random subset of candidate predictors, and then repeatedly splitting the bootstrap sample into subsequent *partitions* based on a candidate predictor that achieves the biggest improvement in a performance metric (by default, the Gini impurity criterion). Trees are typically fully grown in classification, which means that splits are being made until all *nodes* (i.e., the partitions that are subject for a further split) contain no less than one observation, *or* until all candidate predictors have been exhausted for splitting purposes (whichever comes first). A tree-specific prediction for a given observation is then made taking the average over all remaining observations in the terminal node (i.e., a node that cannot be split any further) that corresponds to the observation in terms of predictors. An overall prediction is obtained by taking the average over all tree-specific predictions for the given observation.

RF can determine which variables are important as follows. Each time RF takes a bootstrap sample, it keeps track of which observations that do not fall into in the bootstrap sample (this roughly corresponds to a third of all original observations). These observations are so-called *out-of-bag (OOB)*. A prediction is made for these observations using the tree that was grown on the bootstrap sample, and then an OOB error is estimated. To determine the tree-specific importance of a variable, the variable is *permuted* (i.e., randomly shuffled) in the bootstrap sample, yielding a new variant of the bootstrap sample, and a new tree is grown on the new variant. The new tree then yields a new OOB error, which then is compared with the OOB error of the original tree. The importance of this variable is gauged by taking the OOB error decrease, averaged over all trees, called the mean decrease in accuracy. A large increase in OOB error suggests that the variable is important in predicting the outcome (intention to test for HBV in our case). A ranking of variable importance is made by comparing the increase in OOB errors between variables.

The RF algorithm has several tuning parameters that need to be set by the user. The parameters that are most likely to affect the algorithm’s performance are (1) the number of trees generated and (2) the size of the subset of candidate predictors for each tree. Typically, the values for these parameters are chosen such that performance is optimized. This can be done through cross-validation [2].

We pre-specified the number of trees at 5000 trees, and the random sampled number of variables (mtry) was set at the square root of the total number of variables.

**Interpreting confusion matrices**

The classification accuracy is defined as the number of correct predictions (TP + TN), divided by the total number of predictions (TP + TN + FP + FN).

The sensitivity is the number of persons correctly predicted as having the outcome (TP), divided by the number of persons having the outcome (TP + FN). The specificity is the number of persons correctly predicted as not having the outcome (TN), divided by the number of persons not having the outcome (TN+ FP) [3].

The area under the ROC curve represents the AUC score, which can be interpreted as the probability that a classifier assigns a higher score to a random positive sample compared to a random negative one. Thus, the higher the AUC score, the better the classification result and prediction accuracy.

**Used paper-based questionnaire (translated to English)**

**References**

1. Breiman L. Random forests. Machine learning. 2001; 45(1):5-32.

2. Hastie T, Tibshirani R, Friedman J: The elements of statistical learning 2nd edition. In*.*: New York: Springer; 2009.

3. Florkowski CM. Sensitivity, specificity, receiver-operating characteristic (ROC) curves and likelihood ratios: communicating the performance of diagnostic tests. Clin Biochem Rev. 2008; 29 Suppl 1:S83-7.

**Supplementary tables and figures**

**Table S1. Overview of variables measured by the questionnaire**

| Constructs | Variables | Short variable descriptions | Options |
| --- | --- | --- | --- |
| Intention screening | Intention request  Intention 70 | Intention to request a HBV screening test at the GP on own initiative  Intention to participate in non-refundable HBV screening at € 70,-. | Yes – No |
| Perceived risk | Risk without noticing | Risk having HBV without noticing | Low – High |
|  | Risk infecting someone | Risk of infecting someone with HBV | Low – High |
| Perceived severity of disease | Severity feeling good | HBV is no problem when feeling good | Yes – No – I do not know |
|  | Severity serious disease | HBV is a serious disease | Yes – No – I do not know |
| Stigma regarding HBV | Stigma friends | Others not wanting to be friends when having HBV | Yes – No – I do not know |
|  | Stigma respect | Others less respect when having HBV | Yes – No – I do not know |
|  | Stigma comfort | Others feeling uncomfortable when having HBV | Yes – No – I do not know |
| Shame regarding HBV | Shame others | Feeling ashamed when others know HBV status | Yes – No – I do not know |
|  | Shame guilty | Feeling guilty having HBV | Yes – No – I do not know |
|  | Shame fear | Feeling feared having HBV | Yes – No – I do not know |
|  | Shame disappointment | Feeling disappointed having HBV | Yes – No – I do not know |
|  | Shame not caring | Do not care if others know HBV status | Yes – No – I do not know |
| Perceived  self-efficacy | Self-efficacy | Able to decide HBV screening participation | Yes – No – I do not know |
| Social influence | Social influence friends | Will test myself if friend recommends | Yes – No – I do not know |
|  | Social influence imam | Will test myself if imam recommends | Yes – No – I do not know |
| Perceived benefits | Benefit own health | HBV screening good for my health | Yes – No – I do not know |
|  | Benefit health others | HBV screening good for health others | Yes – No – I do not know |
|  | Benefit clarity | HBV screening gives clarity | Yes – No – I do not know |
| Perceived barriers | Barrier too much time | HBV screening takes too much time | Yes – No – I do not know |
|  | Barrier important | HBV screening not important | Yes – No – I do not know |
|  | Barrier not having symptoms | HBV screening not needed if no symptoms | Yes – No – I do not know |
|  | Barrier trusting Allah | HBV screening not needed only trust Allah | Yes – No – I do not know |
| Knowledge on HBV | Knowledge on HBV | I know nothing about HBV  HBV is an infectious disease  HBV can cause liver cancer  Someone who is looking healthy and feeling good cannot infect others with HBV | True – False |
| Moroccan-Arabic or Berber identity | Moroccan-Arabic or Berber identity | - | Moroccan Arabic – Berber |
| Speaking Dutch | Speaking Dutch | The ability to speak Dutch | Yes – No |
| Knowing someone having HBV | Knowing someone having HBV | Knowing someone having HBV | Yes – No – I do not know |
| Tested for HBV | Tested for HBV | Tested for HBV, self-reported | Yes – No – I do not know |
| Vaccinated against HBV | Vaccinated for HBV | Vaccinated against HBV, self-reported | Yes – No – I do not know |

**Table S2. Performance of the total RF model with ‘Intention request’; confusion matrix**

|  |  | **Observed intention** |  |
| --- | --- | --- | --- |
|  |  | Positive intention | Negative intention |
| **Predicted** | Positive intention | 118 (38.6) | 44 (14.4) |
| **intention by RF** | Negative intention | 63 (20.6) | 81 (26.5) |

The total RF model includes ‘Intention request’ as the dependent variable and all possible determinants as independent variables.

Data are presented as the numbers and percentages of observed and predicted respondents to have a positive or negative intention according to RF.

Performance metrics: ACC: 0.650 (SD: 0.063); AUC: 0.681 (SD: 0.102); SENS: 0.720 (SD: 0.105) and SPEC: 0.572 (SD: 0.136).

**Table S3. Performance of the total RF model with ‘Intention 70’; confusion matrix**

|  |  | **Observed intention** |  |
| --- | --- | --- | --- |
|  |  | Positive intention | Negative intention |
| **Predicted** | Positive intention | 77 (25.4) | 66 (21.8) |
| **intention by RF** | Negative intention | 54 (17.8) | 106 (35.0) |

The total RF model includes ‘Intention 70’ as the dependent variable and all possible determinants as independent variables.

Data are presented as the numbers and percentages of observed and predicted respondents to have a positive or negative intention according to RF.

Performance metrics: ACC: 0.604 (SD: 0.053); AUC: 0.638 (SD: 0.092); SENS: 0.515 (SD: 0.135) and SPEC: 0.669 (SD: 0.121).


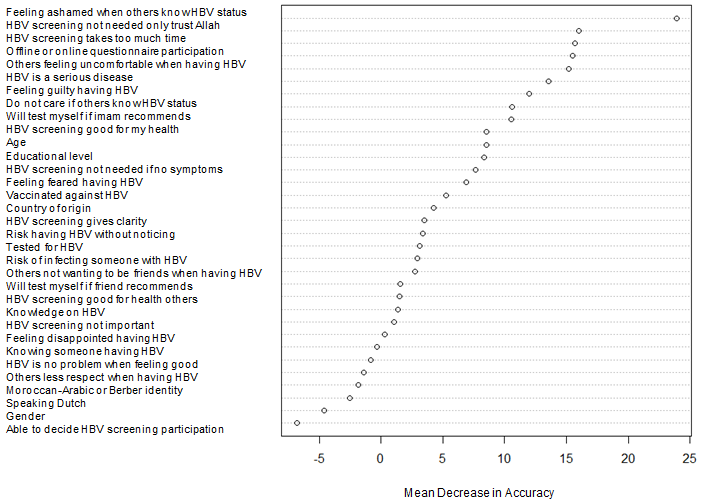


**Figure S1. Variable importance analysis performed by RF for ‘Intention 70’ (n = 303)**

The set of 33 variables used for classification, ordered by their mean decrease in accuracy (importance) as estimated by RF.


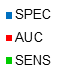

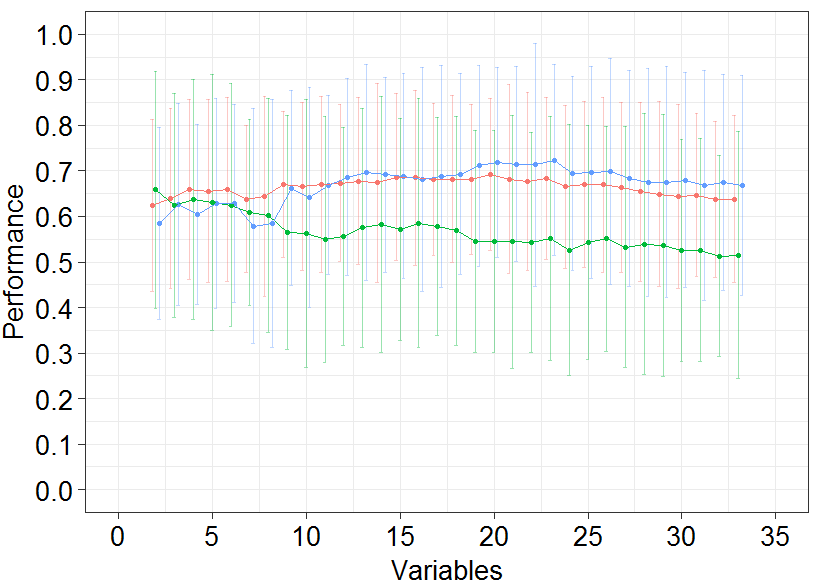


**Figure S2. Result of restricted forward feature selection with RF for ‘Intention 70’**

This figure shows the AUC, SENS, and SPEC for ‘Intention 70’ starting with the most important variable and adding each variable one by one, following the rank obtained through the mean decrease in accuracy (See Figure S1).

**Table S4. Performance of the RF model with the top 10 variables for ‘Intention 70’; confusion matrix**

|  |  | **Observed intention** |  |
| --- | --- | --- | --- |
|  |  | Positive intention | Negative intention |
| **Predicted** | Positive intention | 83 (27.4) | 60 (19.8) |
| **intention by RF** | Negative intention | 58 (19.1) | 102 (33.7) |

Data are presented as the numbers and percentages of observed and predicted respondents to have a positive or negative intention according to RF.

Performance metrics: ACC: 0.611 (SD: 0.016); AUC: 0.666 (SD: 0.092); SENS: 0.563 (SD: 0.147) and SPEC: 0.641 (SD: 0.121).

**Table S5. Marginal probabilities of the top 10 variables in relation to ‘Intention 70’**

| Variables | Content | Answering options | Marginal probability |
| --- | --- | --- | --- |
| Shame others | I would feel ashamed if I have HBV and others would know this | Yes  No  I do not know | 0.420  0.509  0.448 |
| Barrier trusting Allah | Participating in HBV screening is not needed as I only trust Allah | Yes  No  I do not know | 0.471  0.490  0.423 |
| Barrier too much time | Participating in HBV screening takes too much time | Yes  No  I do not know | 0.386  0.496  0.451 |
| Offline or online questionnaire participation | Offline or online questionnaire participation | Offline  Online | 0.497  0.470 |
| Stigma comfort | Having HBV will lead to others feeling uncomfortable having me around | Yes  No  I do not know | 0.418  0.497  0.495 |
| Severity serious disease | HBV is a serious disease | Yes  No  I do not know | 0.490  0.387  0.437 |
| Shame guilty | I would feel guilty if I have HBV | Yes  No  I do not know | 0.444  0.485  0.488 |
| Shame not caring | I would not care if I have HBV and others would know this | Yes  No  I do not know | 0.530  0.477  0.475 |
| Social influence imam | I would have myself tested for HBV if an imam (i.e. Islamic religious leader) would recommend it to me | Yes  No  I do not know | 0.469  0.513  0.478 |
| Benefit own health | Participating in HBV screening is good for my health | Yes  No  I do not know | 0.484  0.464  0.438 |
|  |  |  |  |


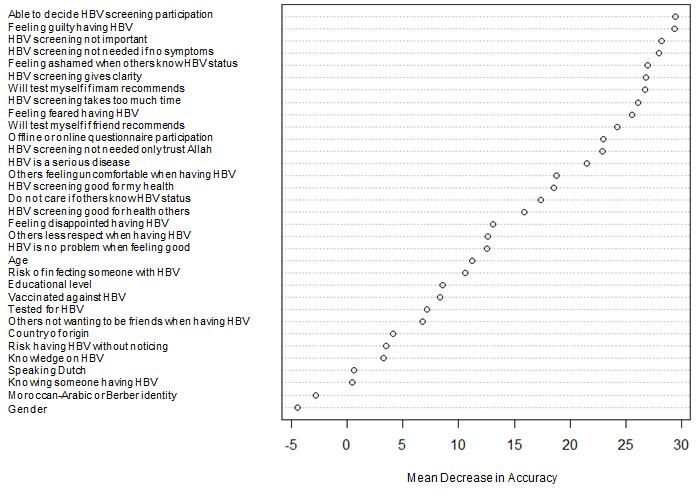


**Figure S3. Variable importance analysis performed by RF for ‘Intention 70’, including missing values as category (n = 379)**

The set of 33 variables used for classification, ordered by their mean decrease in accuracy (importance) as estimated by RF.

**Table S6. Performance metrics of all RF models**

|  | **ACC** | **AUC** | **SENS** | **SPEC** |
| --- | --- | --- | --- | --- |
| **Intention request** |  |  |  |  |
| Total model | 0.650 (SD: 0.063) | 0.681 (SD: 0.102) | 0.720 (SD: 0.105) | 0.572 (SD: 0.136) |
| Restricted model | 0.680 (SD: 0.116) | 0.722 (SD: 0.080) | 0.815 (SD: 0.105) | 0.525 (SD: 0.115) |
| **Intention 70** |  |  |  |  |
| Total model | 0.604 (SD: 0.053) | 0.638 (SD: 0.092) | 0.515 (SD: 0.135) | 0.669 (SD: 0.121) |
| Restricted model | 0.611 (SD: 0.016) | 0.666 (SD: 0.092) | 0.563 (SD: 0.147) | 0.641 (SD: 0.121) |
